# Zebrafish as a Smart Model to Understand Regeneration After Heart Injury: How Fish Could Help Humans

**DOI:** 10.3389/fcvm.2019.00107

**Published:** 2019-08-06

**Authors:** Giorgia Beffagna

**Affiliations:** Department of Cardio-Thoraco-Vascular Sciences and Public Health, University of Padua, Padua, Italy

**Keywords:** myocardial infarction, zebrafish, cardiac regeneration, epicardium, myocardial plasticity

## Abstract

Myocardial infarction (MI) in humans is a common cause of cardiac injury and results in irreversible loss of myocardial cells and formation of fibrotic scar tissue. This fibrotic tissue preserves the integrity of the ventricular wall but undermines pump function, leading to congestive heart failure. Unfortunately, the mammalian heart is unable to replace cardiomyocytes, so the life expectancy for patients after an episode of MI is lower than for most common types of cancers. Whereas, humans cannot efficiently regenerate their heart after injury, the teleost zebrafish have the capability to repair a “broken” heart. The zebrafish is probably one of the most important models for developmental and regenerative biology of the heart. In the last decades, the zebrafish has become increasingly important for scientific research: it has many characteristics that make it a smart model for studying human disease. Moreover, adult zebrafish efficiently regenerate their hearts following different forms of injury. Due to these characteristics, and to the availability of genetic approaches, and biosensor zebrafish lines, it has been established useful for studying molecular mechanisms of heart regeneration. Regeneration of cardiomyocytes in zebrafish is not based on stem cells or transdifferentiation of other cells but on the proliferation of preexisting cardiomyocytes. For this reason, future studies into the zebrafish cardiac regenerative mechanisms could identify specific molecules able to regulate the proliferation of preexisting cardiomyocytes; these factors may be studied in order to understand regulation of myocardial plasticity in cardiac repair processes after injury and, in particular, after MI in humans.

The heart, like any other muscle in the body, requires a constant supply of oxygen, and nutrients to survive. For this reason, there are two large coronary arteries that deliver continuously oxygenated blood to the heart, and if one of these arteries is blocked, a portion of the heart can suffer of "cardiac ischemia” because of the lack of oxygen. If cardiac ischemia lasts long, the heart tissue could die. This condition is known as “heart attack” or “myocardial infarction” or “death of heart muscle.” From a pathological point of view, myocardial infarction (MI) leads to the death of approximately one billion cardiac cells of the left ventricle (about 25% of heart cardiomyocytes) and the irreversible formation of non-contractile fibrotic scar tissue (1). Fibrotic scarring preserves ventricular wall integrity but, at the same time, undermines pump function leading to congestive heart failure (2).

The adult human heart has a very low regenerative capacity on a macroscopic scale after injury; the cardiomyocytes annual renewal rate is estimated between 1% at the age of 25 and 0.45 % at the age of 75 (3). For these reasons, the human heart cannot replace cardiomyocytes lost after MI and, as a result, patients have a lower quality of life and often die prematurely (4). Considering that the one-year mortality rate for MI patients is more than 26%, the life expectancy following an episode of MI is lower than for most common types of cancers, with the exception of lung cancer (5, 6). Medicine has improved prevention and early intervention of MI but it is currently incurable without heart transplantation. Nevertheless, the limited number of donors for heart transplantation does not make this a viable option for most patients. Modern medicine obtained significant advances in the management of the patients after MI with different methodologies: (i) with the use of cardiomyocyte stem cells, that are know to have abilities in generation of new cardiomyocytes, and could be used for repair the injured heart (7–10); (ii) with the reprogramming of fibroblasts into new cardiac cells (11), and (iii) with the production of new biomaterials (12). Although remarkable progress has been made in generating cells of the cardiovascular lineage, a major challenge now is creating engineered tissue architecture to integrate a microvascular circulation. In order to reduce morbidity, and mortality after MI in humans, it is very important to know its regenerative properties, and molecular mechanisms involved in heart regeneration. Hopefully, in the close future, the new knowledge of myocardium biology applied to tissue engineering will make possible to bridge the gap from bench to bedside for a clinically tractable engineered cardiac tissue (12).

The human heart was not always considered a non–regenerative organ. In fact, in the past, it was mostly accepted that the myocardium had some regenerative abilities. It was believed that cardiac hypertrophy was due to the production of new cardiomyocytes, and this idea changed only when different detailed studies demonstrated that pathological cardiac growth was due to increased cardiomyocyte size, and not by cell division (13). Multiple studies have analyzed the mammalian heart after different injuries (14), and these experiments have demonstrated that, in general, the adult mammalian heart does not exhibit the ability to regenerate. However, this classical view has been changed again by fundamental discoveries in the last decade. Porrello et al. demonstrated a transient ability of neonatal mouse heart to regenerate within the first week of postnatal life (15–17). Moreover, other studies performed using stable isotope incorporation during DNA replication have demonstrated that a small number of cardiac cells are renewed during adult life in mammals (18–21). The rate of cardiomyocyte renewal in mammalian adult hearts is clearly not sufficient to compensate for the loss of myocardium after an MI, but the implication resulting from these experiments are promising.

Since the advent of stem-cell biology, the heart has been investigated in order to find stem and/or progenitor cells. The discovery that mammalian adult heart preserves a pool of cardiac stem cells (CSCs) able to participate in cardiac homeostasis and repair opened the field of CSC-based therapy (22). Cell replacement therapy represents a fascinating strategy for myocardial degenerative diseases but despite the encouraging results obtained in small animals, the outcomes from the majority of clinical trials has been poor (10). In the past, CSCs were identified by the expression of c-kit, a typical stemness marker; however, the identification of c-kit positive cell population is necessary but not sufficient in order to define CSCs, because the presence of a heterogeneous population of c-kit positive cells in the heart has been identified. This heterogeneity of c-kit positive cells has probably generated controversy regarding the existence and role of CSCs in the adult heart (23). Recently, different authors have demonstrated that a negative sorting for CD45 and CD31 is necessary to eliminate the lineage-committed cells from the c-kit positive population. At the end, only 1% of the total myocardial c-kit positive cells are really multipotent CSCs (10, 24). The identification of this real multipotent group of cells among positive c-kit cardiac cells could be the key to undertaking new interesting and effective CSCs therapies in the cure of patients after MI.

If the human heart has some endogenous regenerative abilities, these can be supported to promote myocardial regeneration (2). For these reasons, in the last years, scientists have focused their attention on studying natural models of cardiac regeneration. The identification of new therapeutic strategies for regenerating human hearts would reduce morbidity and mortality for millions of people every year. In this review, we will focus on the description of current knowledge about heart regeneration in zebrafish and we will discuss how the information obtained from the study of this interesting model may be used to induce heart regeneration in the adult mammalian heart.

Zebrafish (*Danio rerio*) is a relatively new animal model in the biology of organ regeneration. Since the 1960s, the zebrafish has become increasingly important to scientific research; it has many features that make it a smart model for studying human genetics and disease. Benefits of using zebrafish are: (i) it is small and robust; (ii) it is cheaper to maintain than mice; (iii) it produces hundreds of offspring; (iv) it grows externally, and at an extremely fast rate; (v) it has a short reproductive cycle; (vi) zebrafish embryos are nearly transparent, allowing researchers to easily examine the development of internal structures; (vii) its genome is fully sequenced to a very high quality; (viii) over 70% of human genes have a true ortholog in the zebrafish genome; (ix) as a vertebrate, the zebrafish has the same major organs and tissues as humans; (x) zebrafish have the unique ability to repair heart muscle. Moreover, there are many advantages of using zebrafish to study human disease (25). In particular, essentially due to its willingness to genetic approaches (26), and to the availability of different pathway reporter lines (27–29), zebrafish has been quickly established as a very useful system for studying molecular mechanisms of physiological organ regeneration, including heart (30) (Figure 1).

**Figure 1:**
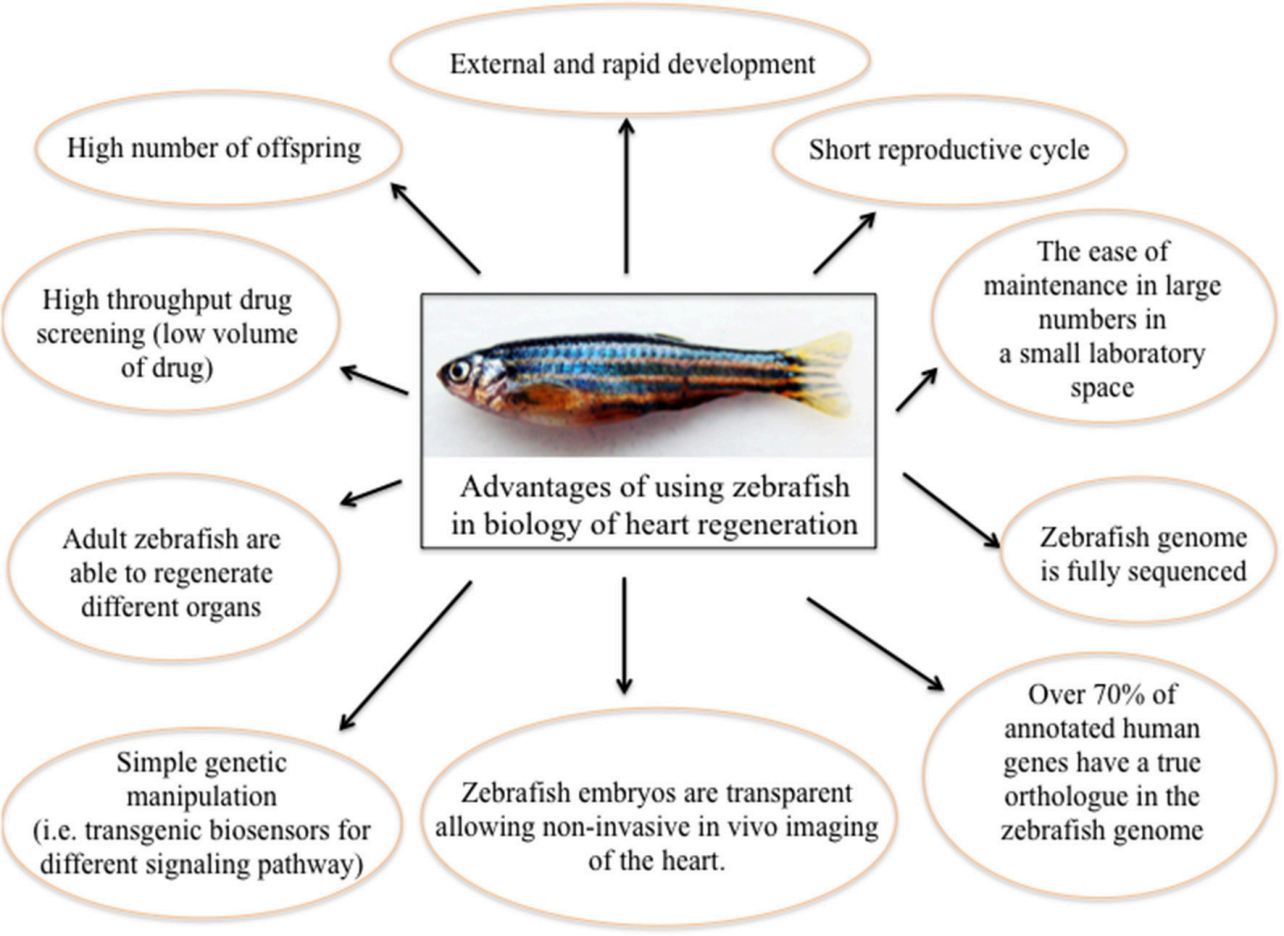
The significance of using zebrafish as a model for heart regeneration.

The zebrafish adult heart has one atrium and one ventricle; it is smaller and simpler than the mammalian heart but the histological and structural composition is very similar to that of other vertebrates. The extraordinary capability of zebrafish *silent heart* (*sih*^*b*109^) mutant embryos of surviving up to 5 days post fertilization thanks to diffused oxygen, in absence of active circulation, leads to considerer zebrafish as the gold standard in the field of developmental cardiovascular research. The identification of multilevel controls that are able to regulate the expression of contractile proteins is fundamental to understanding cardiomyocyte function, dysfunction and regeneration (31). Moreover, more recently new genes required for cardiovascular development, have been identified in zebrafish models through genetic screening strategies (26). For these reasons, zebrafish can be considered an excellent model for study vertebrate development and diseases, but there are some intrinsic disadvantages to the system. Because zebrafish are evolutionarily more distant from humans than murine models, sometimes results obtained from fish experiments will likely have to be confirmed in mammals before being associated to human therapy.

Adult zebrafish are able to regenerate different organs, including all fins (32), the spinal cord (33), the retina (34), the heart (35), the telencephalon (36), and the kidney (37). Interestingly, the mechanisms that control regeneration seem to be organ-specific. For instance, fin regeneration depends on the formation of a structure composed of highly proliferative de-differentiated cells named blastema, able to give rise to all components of the regenerated fin (38). In contrast, regeneration of the telencephalon does not involve the formation of a blastema but requires the activation of a population of cells characterized by the high expression of the Notch target gene *her4.1* (36). Poss et al., in 2002, described for the first time in a zebrafish model the most robust cardiac regenerative response in a vertebrate (35). They demonstrated that zebrafish is able to regenerate its heart after amputation of up to ~20% of its ventricle. The injury leads to the formation of an initial fibrin clot that remains 7–9 days post-injury (dpi); this fibrin clot is replaced by new cardiomyocytes in the following weeks. After 60 dpi the size and shape of the ventricle, as well as the contractile capability of beating heart, gets back to normal (35).

This seminal paper opened a new and challenging field of study regarding cardiac regeneration. Interestingly, as underlined by González-Rosa et al. this study raised many questions that unfortunately were not completely explained in the original paper: (i) why does the zebrafish heart not develop a fibrotic scar? (ii) What are the cellular sources of regenerated tissue? (iii) What signals are involved in regeneration (2)? These questions have been partially clarified in the last 15 years thank to the contribution of different laboratories working in cardiac regeneration field.

In humans, the heart is unable to regenerate the lost cardiomyocytes after MI; instead, the injury triggers the activation of fibroblasts that secrete collagen able to prevent heart rupturing. This collagen-based non-contractile scar persists in the heart and contributes to abnormal systolic function due to its inflexibility, and non-contractibility. Scars formation could eventually lead to heart failure and as consequence it is more a damage than a help to the heart after MI.

The zebrafish has a remarkable capability to regenerate the heart after ventricular injury or amputation, mainly by the ability of the remaining cardiomyocytes to de-differentiate, and proliferate to replace the lost cardiac tissue (35, 39). In 2011 a cryoinjury zebrafish model was generated, which more closely mimics the pathophysiological process experienced by the human heart after MI (40). Different authors demonstrated that the zebrafish heart regenerates after cryoinjury-induced myocardial infarction (41). This model seems to be very interesting because, after cryoinjury, a collagen scar forms at 14–21 dpi, but the zebrafish heart is able to resolve the scar concomitantly to myocardial regeneration, which mammals cannot perform. In zebrafish, after ventricular cryoinjury, cell death, inflammatory infiltration, and increased mechanical forces lead to fibroblasts trans-differentiation into myofibroblasts, and secretion of collagen and ECM components in the wound area. This deposition of ECM components is important in order to maintain the integrity of the cardiac wall following cardiomyocytes death. Progressively, ECM components were degraded by matrix metallopeptidases (MMPs) secreted by cardiac cells and neutrophils. Due to the MMPs important role during cardiac remodeling and end-stage heart failure, better understanding biological function of MMPs in tissue remodeling, and repair after injury in humans remains an essential matter. Among of the MMPs identified to date, MMP-2, and –MMP9 seem to be the main involved in post-MI remodeling. However, the comprehension of MMPs roles is complicated by interactions between different MMPs: sometimes, different MMPs compete each other for the same substrate; moreover, due to compensatory effects, inhibition of a specific MMP can result in the increase of other ones (42). For these reasons, zebrafish could help in the comprehension of pathophysiological MMPs processes post-MI in order to develop novel therapeutic targets able to inhibit specific MMP actions and, as a consequence, to limit the appearance of heart failure post-MI. Particularly, Gamba, et al. demonstrated that, following cryoinjury, transcripts of matrix metalloproteinase genes, *mmp2* and *mmp14*, and Mmp2 enzymatic activity are increased, suggesting the involvement of these proteases in collagen degradation (43).

In literature, it is well-known that in mammals, myocardial infarction-induced fibrosis, and cardiac remodeling are regulated by Smad3-dependent TGFβ signaling (44). More recently, Chablais et al. demonstrated that zebrafish and mammals share similar mechanisms of scar formation. They showed that in zebrafish Smad3-dependent TGFβ signaling is important in the balance between the reparative and regenerative processes and that this signaling is also important for the formation of the transient scar (45).

To explain how the scar is resolved in fish hearts and not in neonatal mouse hearts, Gamba et al. propose a model mechanism of potential scar resolution in zebrafish heart after injury. In the neonatal mouse heart, after cryoinjury, damage leads, at the same time, to the synthesis of collagen, and collagenolytic activity in the wound. The inability to regenerate cardiomyocytes leads to a balance of intra-ventricular mechanical forces and of collagen synthesis and degradation, resulting in both persisting uncontractile collagen scar and in extracellular matrix (ECM) remodeling. In zebrafish heart, the injury leads to a comparable fibrotic response: the intra-ventricular mechanical forces decrease during myocardial regeneration, leading to a down regulation of collagen synthesis and, eventually to the removal of the scar (43). Contraction and generation of mechanical forces are very important for cardiac development and for general cardiac function. Using animal models, researchers have demonstrated that intra-ventricular mechanical forces change with the animal ages, suggesting that tissue composition, such as ECM crosslinking density, and ECM interactions is modified as well (46–48). Moreover, improper mechanical signaling from surrounding tissue, such as the reduction of cardiomyocytes after injury, can lead to the development of defects in the balancing of intra-ventricular mechanical forces and, as a consequence, of collagen synthesis, and degradation (43, 48).

In 2009 Ausoni and Sartore proposed the lack of fibroblasts as an explanation for the regenerative capacity of the zebrafish heart (49). More recently, other authors confirmed the presence of cardiac fibroblasts in the zebrafish heart after injury and revealed that they not only contribute to the fibrotic response but also are necessary for proliferation of cardiomyocyte during heart regeneration (50). In the mouse, cardiac repair upon MI has been demonstrated to happen mainly by ECM deposition from intracardiac fibroblasts, and epicardium. González-Rosa et al. showed that, in the context of heart regeneration, preexisting fibroblasts such as endocardial cells can contribute to collagen production but the main contributor to heart fibrosis is not the endocardium itself. Endocardial cells at the injury edge failed to show a complete fibroblast-like phenotype, probably because they do not undergo full epithelial-to-mesenchymal transition (EMT). Cells from the epicardial border are able to produce both periostin and collagen, whereas, the endocardial cells produced only collagen. This difference in ECM environments surrounding the injury area may play an essential role in heart regrowth (2). Conversely to mammals, in which ECM persists after MI, in the zebrafish heart ECM is degraded. González-Rosa et al. demonstrated that decreased of ECM production by fibroblast have an essential role in fibrosis regression and that this mechanism does not involve the complete elimination of ECM-producing cells: fibroblast are not eliminated but are inactivated (2, 50). Other authors described that, in zebrafish, the limitation of fibrotic response by genetic suppression of *col1a2*-expressing cells compromised cardiomyocytes proliferation. Therefore, they concluded that in regenerative process cells able to produce ECM could be key players (50). This new information regarding how fibrosis influences myocardial regeneration in a species such as zebrafish, with endogenous regenerative potential, could have important implications for future regenerative strategies after MI also in humans; indeed that therapies targeting on fibroblast inactivation could be more efficient than anti-fibrotic ones (2, 50).

Different authors demonstrated that zebrafish regenerate cardiac tissue through the proliferation of pre-existing cardiomyocytes, and neovascularization but, at today, it is not completely clear what signals are involved in zebrafish heart regeneration. After ventricular injury, a blood clot is formed to seal the wound that is subsequently replaced by fibrin, and collagen. Few hours after the injury, the epicardium is activated, and epicardial cells undergoing EMT are able to proliferate and migrate to the injury area. Moreover, Choi et al. showed that FGFs stimulate epicardial cell activation and EMT together with neovascularization during the zebrafish heart regeneration process (51–53).

In the last decade, different authors demonstrated a role of the epicardium, the external epithelial layer of the heart, in myocardial growth through secretion of soluble growth factors (54). When activated, the epicardium secretes different molecules with the ability to regulate heart regeneration. The epicardium has been proposed to stimulate cardiac cells proliferation both in embryonic heart development and adult heart regeneration. The epicardium also stimulates extra cellular matrix (ECM) components able to maintain the integrity of cardiac tissue and electrophysiological properties (55). Huang et al. reported that in zebrafish epicardial IGF signaling is essential for cardiomyocyte proliferation in heart regeneration, suggesting that epicardium could regulate the adult cardiomyocytes developmental gene expression profile during post-injury remodeling of the heart (56) (Figure 2). These data open a new scenario for patients after MI, in which the identification with specific bio-markers of pro-regenerative epicardial cells will support genetic methods able to manipulate precisely only the most appropriate cells after cardiac injury (55).

**Figure 2:**
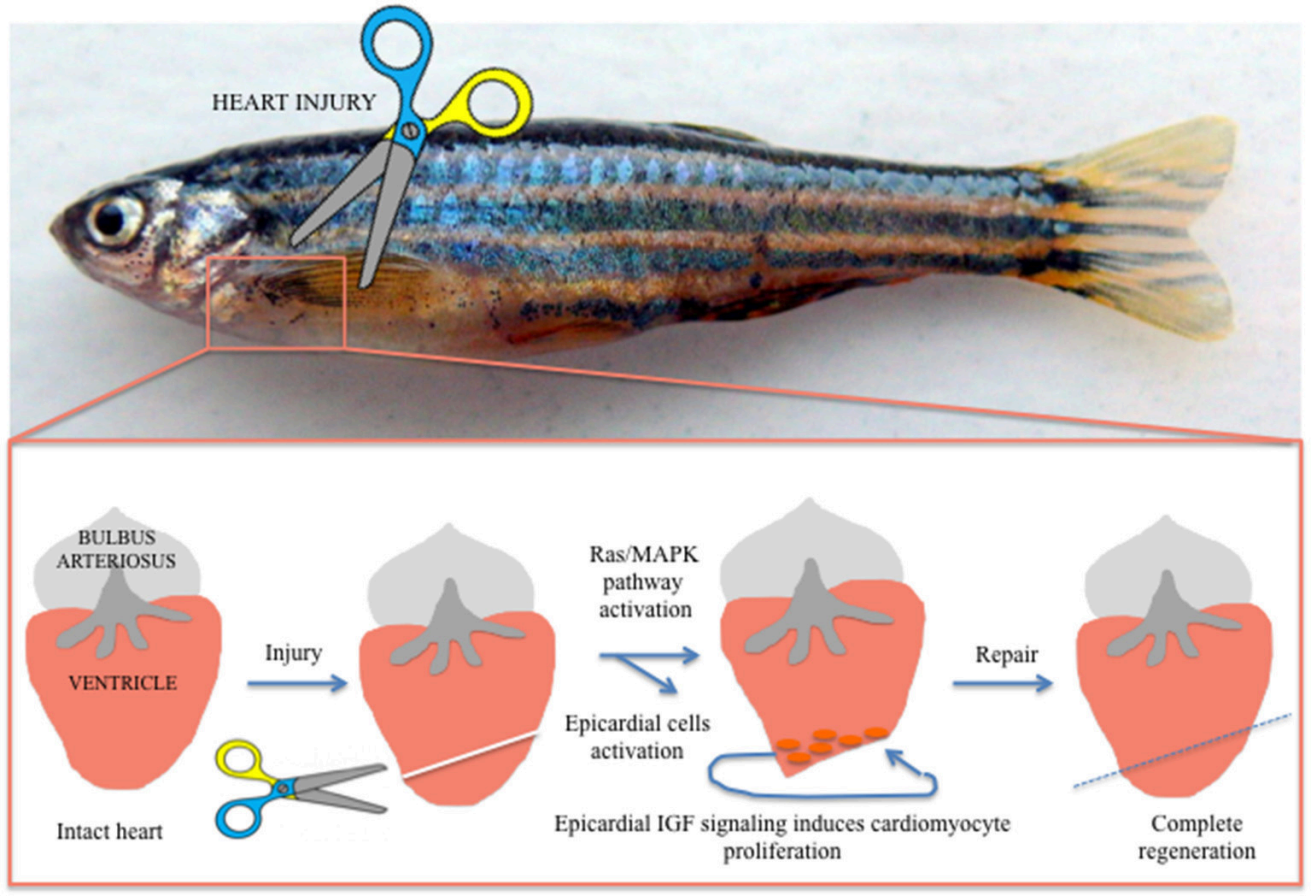
Visualization of heart regeneration in adult zebrafish. After injury the heart activated factors, such as epicardial IGF, able to activate the Ras/MAPK pathway. These activated epicardial cells drive cardiomyocyte regeneration after injury and are essential for cardiomyocyte proliferation in heart regeneration.

To date, only few information is known about the signaling pathways underlying cardiac regeneration in zebrafish. Different studies demonstrated that the molecular mechanisms able to drive cardiomyocyte proliferation after injury involve ligands such as fibroblast growth factors (FGFs) (53), transforming growth factor β (TGFβ) (45), platelet derived growth factor β (PDGFβ) (57), insulin-like growth factor (IGF) (56) Neuregulins (Nrg) (58), and bone morphogenetic proteins (BMPs) (59).

The majority of these factors activate the Ras/MAPK pathway, which is controlled by an ERK phosphatase named feedback attenuator dual specificity phosphatase 6 (Dusp6). Missinato et al. showed that suppressing Dusp6 function, either by small molecules such as BCI and BCI215 or by gene inactivation, enhances zebrafish heart regeneration within 4–7 dpi but not beyond 12 dpi (60). Importantly, this effect was observed after cardiac amputation but not in uninjured hearts, implying that the effects of these compounds are injury-dependent. It will be interesting to determine whether BCI and BCI215 could have the same increased proliferation effect on cardiomyocytes also in other injury models, such as cryoinjury or cardiomyocyte ablation (45, 61–63), or in the neonatal mouse (64).

The ability of mammals to repair the heart is a very limited event but the identification of signaling pathways able to enhance cardiac proliferation could be used in order to promote mammals repair ability. One molecule that is fundamental in regulating cardiomyocyte proliferation in both zebrafish and mice is Neuregulin 1 (Nrg1), a cell adhesion molecule essential for the normal development of the nervous system, and heart. Recent findings show that Nrg1 can stimulate heart repair. Blocking Erbb2, the Nrg1 co-receptor, using the chemical inhibitor AG1478 restricts cardiomyocyte proliferation in zebrafish heart regeneration after injury. So BCI could be used in mammals to enhance Nrg1 signaling. Unfortunately, different trials showed that Nrg1 therapies induce tumor formation (65). However, a recent study performed using mice as model organism showed the absence of neoplastic growth after Nrg1 administration (66). Nevertheless, the idea of the use of lower concentrations of Nrg1 together with chemical inhibition of Dusp6 could be used to stimulate cardiomyocyte proliferation for cardiac repair (60).

Mammals and lower vertebrates adult cardiomyocytes have significant difference in proliferative capacity, probably due to ontogenetic, and/or phylogenetic factors. Understanding these factors could be useful for the development of novel therapeutic strategies that encourage cardiomyocyte proliferation. In the last years, different molecular pathways are under investigation for their potential ability to influence cardiomyocyte proliferation both in mammals and fish: hippo/YAP/TAZ, Meis1, Wnt/β-catenin, IGF, Ros, TGFβ-activin, Hypoxia, Monocyte/macrophage, CDK9/PTEFb, and miRNA (67). The Hippo/Yap/Taz pathway seems to be important in enhancing cardiac regeneration; this pathway plays an important role both in the heart development and in postnatal cardiomyocyte proliferation. IGF2 has been demonstrated to be able to activate cardiomyocyte proliferation and is required for heart regeneration in zebrafish, whereas TGF β/activin signaling seems to be a key regulator in cardiomyocyte proliferation and scar formation. Puente et al. have demonstrated that in adult cardiomyocytes cell cycle arrest could be triggered by mitochondrial reactive oxygen species-mediated oxidative DNA damage, and as a consequence, hypoxia, and redox signaling also could be regulators of cardiac renewal (68). This suggests that, to proliferate efficiently, cells responsible for cardiomyocytes renewal, such as immature myocytes or progenitor population, could need an environment with a lower concentration of oxygen. At the end, also miRNAs seem to be important for cardiomyocyte proliferation. Different authors demonstrated that miRNAs are able to affect cardiomyocytes proliferation by inhibiting or activating the cell cycle. Interestingly, the role of some of these pathways in mammal cardiomyocyte proliferation has been identified using zebrafish as model for cardiac repair studies (67).

Future studies into the zebrafish cardiac regenerative mechanisms could identify specific molecules able to regulate heart regeneration; these molecules may be used to understand how myocardial plasticity can be maintained during regeneration in order to promote cardiac repair after MI also in humans. Moreover, the identification of factors that trigger heart regeneration could not be enough to completely understand how myocardial plasticity is regulated after heart injury or MI. Signaling pathway networks and epigenetic regulation represent intriguing factors to be analyzed in future studies to understand how myocardial plasticity is blocked and reactivated. In this perspective, also for these analyses of cardiac plasticity zebrafish can be used as a preclinical model, useful to identify new therapeutic strategies to reduce the damages associated with MI.

## Author Contributions

The author confirms being the sole contributor of this work and has approved it for publication.

### Conflict of Interest Statement

The author declares that the research was conducted in the absence of any commercial or financial relationships that could be construed as a potential conflict of interest.
